# Dual-Wavelength 980 nm and 1550 nm Laser Therapy Accelerates Alveolar Socket Healing After Tooth Extraction

**DOI:** 10.3390/dj14010017

**Published:** 2026-01-01

**Authors:** Dinislam Davletshin, Aglaya Kazumova, Alexey Fayzullin, Nune Vartanova, Peter Timashev, Andronik Poddubikov, Svetlana Tarasenko, Pavel Kryuchko, Ivan Klenkov, Petr Panyushkin, Mikhail Nelipa, Marina Skachkova, Ekaterina Diachkova

**Affiliations:** 1 Department pf Oral Surgery, Borovskiy Institute of Dentistry, Sechenov University, 8/2 Trubetskaya St., Moscow 119991, Russia; davletshin.dinislam@gmail.com (D.D.); aglaya.kazumowa@yandex.ru (A.K.); tarasenko_s_v@staff.sechenov.ru (S.T.); petukhova_m_m@staff.sechenov.ru (M.S.); 2 Institute for Regenerative Medicine, Sechenov University, 8/2 Trubetskaya St., Moscow 119991, Russia; fayzullin_a_l@staff.sechenov.ru (A.F.); timashev_p_s@staff.sechenov.ru (P.T.); 3 Laboratory of Microbiology of Opportunistic Bacteria, I.I. Mechnikov Research Institute for Vaccines and Sera, 5a Malyi Kazenniy Pereulok, Moscow 105064, Russia; labmicr@mail.ru; 4 Sklifosovskiy Institute of Clinical Medicine, Sechenov University, 8/2 Trubetskaya St., Moscow 119991, Russia; andronikpoddubikov@gmail.com; 5 Department of Operative Surgery and Topographic Anatomy, Sechenov University, 8/2 Trubetskaya St., Moscow 119991, Russia; kryuchko_p_v@staff.sechenov.ru (P.K.); panyushkin_p_v@staff.sechenov.ru (P.P.); nelipa_m_v@staff.sechenov.ru (M.N.)

**Keywords:** alveolitis, laser therapy, medical laser, surgical dentistry, dentistry, regeneration

## Abstract

**Background/Objectives**: Alveolitis, or “dry socket,” is a common complication after tooth extraction, associated with pain, inflammation and delayed healing. Standard surgical treatments are often invasive and insufficient. Laser therapy offers antimicrobial, anti-inflammatory and regenerative effects. This study aimed to compare the efficacy of 980 nm monolaser therapy and 980 nm and 1550 nm dual-wavelength therapy on alveolar socket healing in a rabbit model. **Methods**: In vitro tests evaluated bactericidal effects of 980 nm laser exposure. Eighteen adult male chinchilla rabbits underwent the extraction of the first incisors with the prevention of clot formation to model alveolar socket healing. On day 3, animals were randomized to three groups: mechanical curettage and antiseptic irrigation, 980 nm diode laser therapy, or combined 980 nm + 1550 nm therapy. Clinical parameters (hyperemia, edema, pain, socket closure) were assessed up to day 7. Histological and microbiological analyses were performed on days 7 and 12. **Results**: Laser therapy showed superior outcomes compared to mechanical treatment. In vitro, 980 nm exposure eradicated microorganisms after 3 s. By day 7, hyperemia decreased to 0.7 ± 0.6 points in the dual-laser group, versus 2.0 ± 0.0 (980 nm) and 3.0 ± 0.0 (mechanical). Complete socket closure occurred in 33% with mechanical treatment and in 67% of sites in the dual-laser group. Pain was fully resolved only after dual-laser therapy. Histology confirmed more organized granulation tissue and angiogenesis in the dual-laser group. **Conclusions**: Dual-wavelength laser therapy demonstrated superior anti-inflammatory, antimicrobial and regenerative effects compared with diode monotherapy and mechanical treatment. These findings highlight its promise as a minimally invasive approach for managing alveolitis, warranting further clinical evaluation.

## 1. Introduction

Alveolitis, or dry socket, is one of the most common and painful complications arising after tooth extraction. Its pathogenesis is associated with the disruption of the formation and subsequent disintegration of the blood clot within the alveolus, leading to exposure of the bone walls and the development of intense inflammation. The prevalence of this pathology ranges from 0.5% to 5% after routine extractions, reaching 30% or more after the removal of impacted lower third molars, significantly reducing patients’ quality of life and prolonging rehabilitation [1].

The standard treatment protocol for alveolitis includes curettage of the socket to remove necrotic tissue, irrigation with antiseptic solutions, and application of topical anti-inflammatory and antibacterial agents. Despite their widespread use, these methods have several significant limitations: they are invasive, do not completely eradicate pathogenic microflora, and do not stimulate active regenerative processes. Furthermore, mechanical treatment can further traumatize the socket walls and slow healing. Therefore, a pressing challenge in modern dentistry is the search for more effective and minimally invasive treatment methods [2].

In recent decades, laser technologies have attracted significant interest from physicians. Laser therapy offers new perspectives in the treatment of inflammatory diseases of the oral cavity due to its precision and minimally invasive action, disinfectant effect, ability to modulate cellular metabolism, and stimulate regeneration [3]. In particular, diode lasers with a wavelength of 980 nm demonstrate high efficiency based on the optimal absorption in hemoglobin and water, which ensures a good hemostatic and ablative effect while gently affecting surrounding tissues. It is believed that their use in dentistry can provide effective antisepsis and also relieve inflammation, reduce pain, and accelerate epithelialization and granulation tissue formation [4]. However, despite the encouraging results of individual pilot studies, the evidence base supporting the widespread use of diode lasers for alveolitis remains insufficient [5,6,7].

The aim of this study was to compare the effects of 980 nm monolaser therapy and 980 nm and 1550 nm dual-wavelength therapy on alveolar socket healing and the reduction in complications. To achieve this goal, we conducted a clinical assessment of the condition of postoperative sockets based on the dynamics of hyperemia, swelling, pain syndrome, and the rate of soft tissue regeneration. The biopsies were subject to a histological analysis to determine whether laser treatment modulated inflammation or regeneration. A microbiological study of the socket contents was conducted to identify the optimal laser mode with a wavelength of 980 nm, determine the composition of the microbial flora, and comparatively evaluate the antibacterial effectiveness of the laser therapy.

## 2. Materials and Methods

### 2.1. Study Design

All procedures complied with international animal welfare standards and were approved by the local ethics committee (protocol no. 09-24 dated 3 April 2024). The study on laboratory animals was conducted in the main vivarium of Sechenov University and the Department of Surgical Dentistry of the E.V. Borovsky Institute of Dentistry of Sechenov University. The source of animals was the Federal State Budgetary Institution “VGNKI” “Manikhino” (Istra, Moskovskaya oblast, Russia).

The sample size calculation assumed a type I error probability of 5% (*p* = 0.05) and a type II error probability of 10% (the study’s power was 90%). Taking into account the above criteria for sample calculation, the minimum number of observations was 6 tissue samples for each experimental group for each time point (18 tooth extraction sockets for day 7, and 18 for day 12). The calculation was performed using the ClinCalc.com sample size calculation formula.

**Figure 1:**
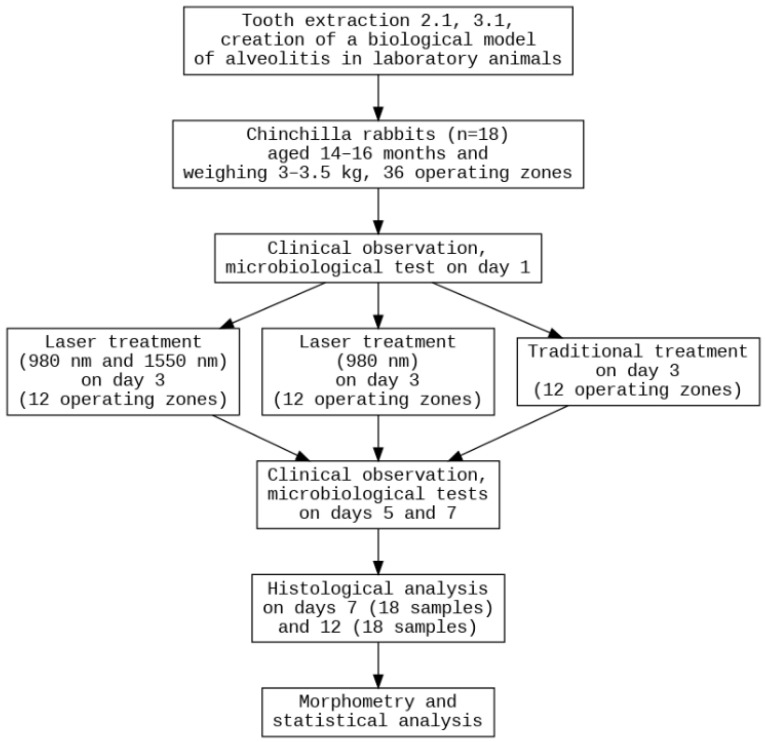
Study design. Impaired alveolar socket healing was induced by extracting teeth 2.1 and 3.1 in 18 rabbits (36 sockets in total). A two-level randomization strategy was applied: first, animals were assigned to two endpoint groups (days 7 and 12), and second, individual sockets were randomized to treatment modalities. Nine rabbits (18 sockets) were allocated to each time point. For every endpoint, six sockets per treatment group were included: conventional management, 980 nm laser therapy, and combined 980 + 1550 nm laser therapy. Clinical monitoring and macroscopic assessment were performed through day 12. Microbiological samples were obtained on days 1, 5 and 7, and histological analyses were carried out at the designated endpoints (days 7 and 12).

The experiment involved 18 healthy, adult male Chinchilla rabbits, aged 14–16 months and weighing 3–3.5 kg (Figure 1). The animals were maintained at a temperature of 18–22 °C, humidity of 50–65%, with a 12-h light cycle and 10 air changes per hour. The rabbits were housed in individual cages with wood bedding, which was changed daily. Their diet consisted of standard compound feed and filtered water. Housing conditions complied with Directive No. 63 of the European Parliament and Order No. 267 of the Russian Ministry of Health. No rabbits of female gender, without prior immunosuppression, with weight less than 3 kg or over 3.5 kg, different breeds or without quarantine clearance were included in the study. The main exclusion criterion was a lethal outcome, which can occur after surgical operations involving sedation. Each study group included six sockets obtained from six different rabbits, with three sockets originating from the mandible and three from the maxilla. Because the experiment involved two evaluation time points (days 7 and 12) and three treatment modalities, the total number of animals required for each time point was nine.

The primary endpoint was the reduction in inflammation findings severity on postoperative day 7, expressed as the scores of hyperemia, edema and pain. Secondary endpoints included (1) defect closure rate and granulation tissue thickness from histomorphometric analysis and (2) reduction in microbial load (CFU/mL) in the socket.

### 2.2. Laser Device Characteristics

Alta-Soyuz surgical laser device (registration certificate for a medical device dated 12 January 2021 No. 2020/13139, VPG LaserOne, Moscow, Russia) was used as the basis of a diode laser with a wavelength of 980 nm (radiation delivery via an optical fiber-contact effect), and a fiber laser with a wavelength of 1550 nm (non-contact effect on tissue-fractional laser treatment). The wavelength of 980 nm is used in laser therapeutic dentistry (treatment of aphthous ulcers, herpes and other oral mucosal diseases), endodontics, periodontitis treatment, aesthetic dental surgeries (gum recession, gingivoplasty), contact surgery of oral soft tissues (inflammatory oral mucosal diseases, frenectomy, operculectomy), and implantation. Therapy with a wavelength of 1550 nm is used in gum biotype modification surgeries, cyst sclerotherapy, recession closure, oral mucosal depigmentation, scar smoothing, and gum keratinization.

These devices offer high precision, minimal invasiveness, and the ability to accelerate regeneration processes, making them promising for the treatment of alveolitis.

The laser therapy parameters are listed separately for 980 nm and 1550 nm lasers in the following paragraphs. The peak output power, spot size, duty cycle, repetition rate and fiber size were taken from the technical manuals. 

Wavelength 980 nm: peak output power 25 W, beam divergence 0.4 ± 0.1 rad, power density 19.8 kW/cm^2^, spot size/beam diameter on tissue 0.4 mm, fluence 198 kJ/cm^2^, duty cycle 10%, repetition rate 10 Hz, fiber core size 365 µm. Exposure time: 2 s (for animal studies), 1, 2, 3 s (for microbiological studies). Spot area: 0.00126 cm^2^. Power density: 19.8 kW/cm^2^. Pulse duration: 10 ms. Flux density: 198 J/cm^2^. The 980 nm laser fiber was placed in direct contact with the wound surface and moved across the socket in an elliptical trajectory over a 2-s exposure.

Wavelength 1550 nm: output peak power 1.5 W, beam divergence 0.4 ± 0.1 rad, power density 530 W/cm^2^, spot size/beam diameter on tissue 0.6 mm, fluence/energy 0.053 J/cm^2^, pulsed mode (SP), extremely low duty cycle (0.1%), repetition rate 10 Hz, irradiation distance for “non-contact” mode 3 mm, fiber core size 600 µm. Exposure time is 3 s. Spot area: 0.00283 cm^2^. Power density: 530 W/cm^2^. Pulse duration: 0.1 ms. Flux density: 0.053 J/cm^2^. The nozzle optics are fraxel (focusing). The 1550 nm laser fiber was positioned 3 mm above the socket using a fixed spacer at the fiber tip, and moved in an elliptical trajectory over the surface for 3 s.

The manufacturer performs calibration every two years. Our laser device was last calibrated on 10 April 2024, one week before the experiment began, and remained stationary throughout the entire study period.

### 2.3. Surgical Technique

Before the procedure, the rabbits were given a 24 h fast. The animals were anesthetized intramuscularly (tiletamine and zolazepam, Vesotil, at a dosage of 5 mg/kg body weight) and locally (articaine without adrenaline, at a dosage of 0.1 mL/kg). Xylazine (Xyla, at a dosage of 0.2 mL/kg) was used to provide muscle relaxation. Extraction of teeth 2.1 and 3.1 in laboratory animals involved cutting the annular ligament of the tooth using a sickle-shaped spatula and a scalpel, which was passed around the gingival pocket to the bone tissue. Tooth extraction was performed with extraction forceps, which grasped the tooth crown as low as possible to reduce the risk of fracture.

The sockets of extracted teeth were temporarily filled with cotton swabs for 15 min to prevent blood clot formation and simulate alveolar socket healing. This created conditions similar to complications encountered in clinical practice in patients with alveolitis. On day 3, alveolitis was diagnosed in all animals according to the macroscopic characteristics of this disease.

The rabbits were randomly assigned to the two planned observation time points (day 7 and day 12) using a computer-generated randomization list. A second level of randomization was then applied to allocate individual mandibular sockets to one of the three experimental groups. Each socket was pre-labeled with a unique alphanumeric code (one letter + one number), and group assignment was performed. This two-step randomization ensured that both the time point and the treatment allocation were unbiased. Full blinding was implemented for microbiology analysis. A morphological laboratory researcher (A.F.) received information about the anatomical location, group assignment and treatment modality for descriptive analysis; however, he used intralaboratory sample coding and a universal protocol for morphometry.

**Figure 2:**
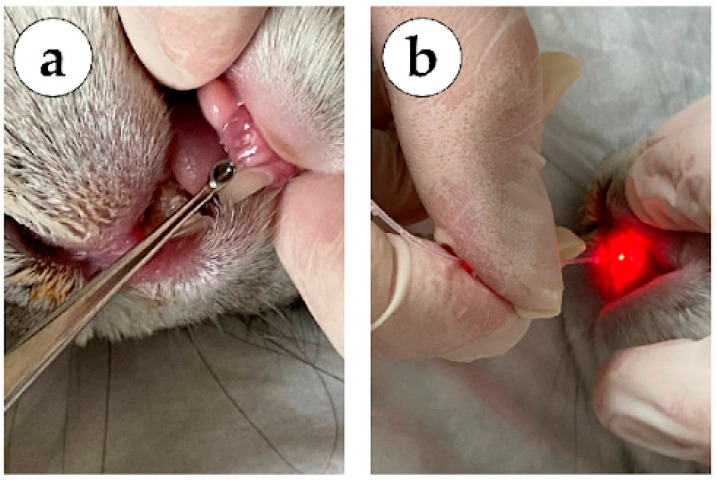
Surgical procedure. (**a**) Curettage of the socket of the extracted tooth of the lower jaw (3.1) using the mechanical method. (**b**) Treatment of the socket of an extracted tooth of the upper jaw (2.1) with a diode laser (980 nm).

In group 1 (mechanical methods, no laser treatment), the sockets of the extracted teeth were treated on day 3 using surgical methods: curettage of granulations with a dental curettage spoon (lower jaw tooth on Figure 2a), subsequent treatment of the socket of the extracted tooth with a 0.05% Chlorhexidine antiseptic solution, installation of a hemostatic sponge to protect the wound surface.

In group 2 (980 nm laser), the sockets of the extracted teeth were treated on day 3 using a dental laser system (Alta-Soyuz with a wavelength of 980 nm) for 3 s in a spiral (upper jaw tooth on Figure 2b), over the entire area of the socket of the extracted tooth (contact interaction).

In group 3 (laser 980 nm + 1550 nm), the sockets of the extracted teeth were treated on day 3 using a dental laser system (Alta-Soyuz, IRE-Polis, Moscow, Russia) with a wavelength of 980 nm) for 3 s in a spiral, over the entire area of the socket of the extracted tooth (Figure 2b), and a dental laser system (Alta-Soyuz with a wavelength of 1550 nm) on the mucous membrane of the oral cavity, along the periphery of the socket of the extracted tooth for 2 s (non-contact interaction).

Animals received adequate pain relief therapy in the postoperative period (0.03% ketarolac, Biosynthez, Penza, Moscow, Russia), 0.1 mL intramuscularly after tooth extraction-twice a day for a three-day period.

The condition of the defects was assessed macroscopically on days 1, 3, 5 and 7. The hyperemia was assessed on a 4-point scale (0—normal color, 1—mild hyperemia, 2—moderate hyperemia, 3—bright hyperemia). Edema was measured on a 4-point scale (0—none, 1—slight, 2—moderate, 3—severe). Pain was assessed using the Rabbit Grimace Scale (RbtGS) [8]. The defect sizes were measured as the average between two perpendicular diameter measurements, width and length, on days 7 and 12 of the experiment.

In order to exclude the impact of tooth location (upper of lower jaw) on the macroscopic features of inflammation, a comparative analysis was conducted. A comparison of hyperemia, swelling and pain between the upper and lower sockets of the same rabbit across all observation days revealed no consistent significant differences. In most cases, the values in paired sockets of the same rabbit were either identical or differed only slightly (usually by no more than 1 point on the appropriate scale). For example, on day 1, in control animals (without laser), hyperemia in the upper socket was rated, on average, 0.17 points higher than in the lower socket (2 points vs. 1 point in some rabbits), while in other rabbits, the lower socket sometimes had a slightly higher score. Similarly, by day 3, in the same group, hyperemia scores had equalized between the sockets. In the laser therapy groups (980 nm and combined 980 + 1550 nm), no significant imbalance was observed: at most time points, the differences between the upper and lower sockets were absent or around 0.5 points and were not statistically significant.

A similar pattern was observed for edema. Minor differences between sockets were observed at the earliest stages: for example, in the control group, on days 1–3, the lower socket in some rabbits had slightly more swelling than the upper socket (a difference of up to 1 point). However, this trend was not maintained in other animals; greater swelling was observed in the upper socket, or the swelling was equal in both sockets. By day 5, swelling in the upper and lower sockets was virtually identical in all rabbits in all groups; the differences were not significant. On the remaining days (days 7 and 12), swelling was either absent or minimal and equal in both sockets for each rabbit, regardless of treatment method.

Regarding pain, it was initially assessed as a single total score for each rabbit (therefore, in the original table, this score was duplicated for the upper and lower sockets). Thus, pain scores were consistent for paired sockets for each rabbit on all days, and for one animal, no differences were observed between the upper and lower sockets. This confirms that the pain score was related to the overall condition of the rabbit, not to a specific socket.

No exclusions, missing data, or adverse events occurred during the study, and all animals completed the planned procedures and follow-up assessments.

### 2.4. Histological Analysis

The oral cavity tissues were fixed in 10% neutral buffered formalin (Biovitrum, Moscow, Russia). The samples were decalcified in electrolytic solution (Biovitrum, Moscow, Russia) over two months, sectioned in cross-sectional orientation, perpendicular to the mandibular surface, dehydrated in isopropyl alcohol in Epredia STP120 spin tissue processor (Thermo Fisher Scientific, Waltham, MA, USA) and embedded into paraffin blocks using HistoStar embedding workstation (Thermo Fisher Scientific, USA). No significant tissue loss occurred during decalcification or sectioning; all specimens remained intact for analysis. 4 μm thick sections were obtained with Leica RM 2125RTS microtome (Leica Microsystems, Wetzlar, Germany) and stained with hematoxylin and eosin (Biovitrum, Russia) and Picrosirius red (Abcam, Cambridge, UK). The slides were digitized at ×400 magnification mode using NanoZoomer S20MD scanner (Hamamatsu, Saitama, Japan) for morphological analysis. The thickness of the granulation tissue layer between the detritus and alveolar bone was evaluated as an average of 5 measurements at a distance of 200 μm from each other. All measurements were performed by a pathologist blinded to the experimental group assignment of each sample.

### 2.5. Microbiological Analysis

The samples were collected from sockets without prior irrigation. The in vitro microbiological study was conducted using 9 test tubes (1 mL), each containing a suspension of microorganisms (1.5 on the McFarland scale, Streptococcus mutans, Staphylococcus aureus, Candida albicans), to determine the optimal laser mode with a wavelength of 980 nm (exposure time—1, 2 and 3 s). After exposure of the suspensions to lasers, the test tubes were processed for quantitative microbiological analysis.

For the in vivo study, samples were collected from the socket with swabs and a sterile container (ABS plastic, Qingdao, China) on days 1, 3, 5 and 7, and processed for subsequent cultivation (Interscience easySpiral Pro Milk, Saint Nom la Bretèche, France) on an Agar medium (Agar with brain heart extract, Condalab (Madrid, Spain)), analysis of the socket content of the extracted tooth (MALDI Biotyper^®^ sirius mass spectrometer, Bruker, Bremen, Germany) and quantitative microbiological analysis (Interscience Scan 4000 automatic colony counter, Saint Nom la Bretèche, Paris, France).

The temperature of the microbial suspension was measured using a contact thermometer with an immersion probe inserted directly into the solution. A maximum temperature of no higher than 42 °C was considered acceptable. Irradiation regimes that demonstrated a reduction in microorganism counts at solution temperatures ≤ 42 °C were considered potentially suitable for further use while maintaining an adequate thermal exposure.

The preparation and dispensing of nutrient media were performed using a MEDIAWEL 10 automatic media cooker and a DISTRIWEL 440 dispensing module (Bruz, France). Two successive 100-fold dilutions in physiological NaCl solution were prepared from the delivered material. All dilutions, including the original samples, were plated on Petri dishes with the appropriate nutrient agar. The cultivation was performed in parallel on three types of media: agar with brain–heart infusion with 1% arapa (HighMedia Laboratories Pvt. Ltd., Thane, India); agar with brain-heart infusion with the addition of (5%) horse defibrinated blood (JSC ECOlab, Moscow, Russia); Sabouraud’s medium for fungal isolation (State Hydrological Institute, St. Petersburg, Russia). The bacterial suspension was cultivated in a spiral mode in a volume of 50 μL using the easySPIRAL Pro automatic cultivation station, Intersciens (Saint Nom la Bretèche, France), onto Mueller Hinton Agar plates (HiMedia, Thane, India). The plates were incubated at 37 °C in a normal atmosphere for 24 h (Thermo B-20 Thermostat-Incubator, Stockholm, Sweden), with blood agar at 37 °C in an atmosphere with 5.5% CO2 for 24 h (MCO-15AC Incubator, Sanyo Electric Co., Ltd., Osaka, Japan), with Sabouraud in a normal atmosphere at 32 °C for 48 h. The results were recorded using a Scan4000 automatic colony counter, Intersciens (France). Colonies were counted for each dilution, and the result obtained was multiplied by the dilution factor. Pure cultures of microorganisms were obtained. Microbial cultures were identified using MALDI-TOF mass spectrometry on a MALDI Biotyper Sirius RUO System (Bruker, Bremen, Germany). The identification result was considered reliable if the database match coefficient (Score) was greater than or equal to 2.0.

### 2.6. Statistical Analysis

The statistical analysis of the experimental data was performed with a standard program package, GraphPad Prism version 10.00 for Windows (GraphPad Software, Inc., San Diego, CA, USA). The normal distribution of the quantitative data was checked by Shapiro–Wilk’s normality test. The intergroup differences in defect size, granulation tissue thickness and microbiology results were analyzed via one-way ANOVA followed by Tukey’s multiple comparison test; the results were presented as column graphs of the mean values ± SD. Differences in scores were assessed using the Kruskal–Wallis test with Dunn’s multiple comparison test; the results were presented as column graphs with median values and interquartile range CI. The Friedman test was applied for repeated measurements. *p*-values equal to or less than 0.05 were considered statistically significant.

## 3. Results

### 3.1. Macroscopic Evaluation

**Figure 3:**
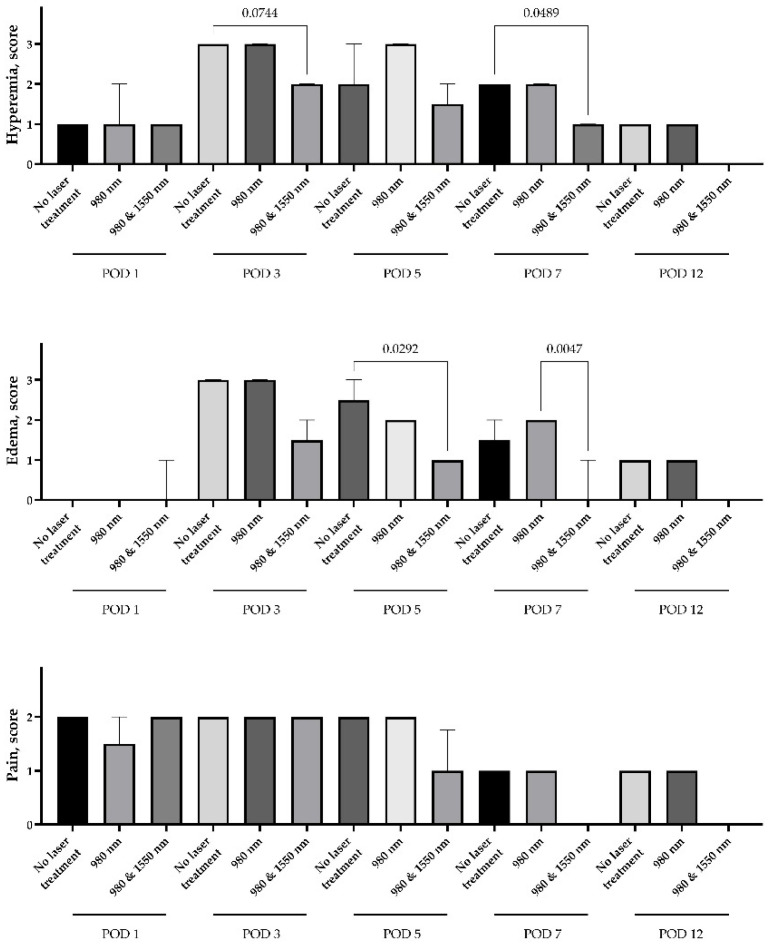
Dynamics of clinical markers of inflammation after treatment of alveolitis, Kruskal-Wallis test with Dunn’s multiple comparison test, median values ± interquartile ranges, *p* values less than 0.2 are presented on the graph.

The use of both 980 nm monotherapy and a combination of 980 nm and 1550 nm demonstrated greater efficacy in relieving alveolitis symptoms compared to treatment without lasers. There was no difference in hyperemia, edema or pain between the study groups on Day 1 of the experiment. Hyperemia and edema markers were elevated by Day 3, however, hyperemia in groups treated with dual-wavelength therapy had a trend to be lower than in the control or mono laser therapy group (*p* = 0.07 and 0.15, subsequently). The differences in hyperemia (*p* = 0.049) and edema (*p* = 0.005) persisted until Day 7 of the experiment. Interestingly, there was a nonsignificant rise in edema after mono laser therapy compared to the control without lasers; however, the difference between mean scores was only 0.5 (Figure 3).

The severe pain syndrome persisted for over 3 days in all study groups. On Day 5, there was a trend for a 0.75 score decrease in the dual-wavelength group compared to the control and mono laser groups (*p* = 0.16). Since Day 7, the pain was not registered even at the level of score 1, in any rabbit of the dual-wavelength group. However, the mild score 1 pain was registered in rabbits of other groups on Days 7 and 12.

Correlations were calculated between the degrees of pain, swelling and hyperemia for all observations. Reliable positive relationships were found between all three clinical indicators: thus, the swelling score correlated significantly with the hyperemia score (Spearman’s correlation coefficient r_s_ ≈ 0.73, *p* < 10^−27^), as well as with the pain level (r_s_ ≈ 0.57, *p* < 10^−14^). The correlation between pain and hyperemia was also highly significant (r_s_ ≈ 0.63, *p* < 4 × 10^−18^). These results indicate that more pronounced inflammation (tissue redness and swelling) usually accompanies more severe pain, which is quite natural. The most significant relationship was noted between hyperemia and swelling of the socket. The relationship between pain and objective signs of inflammation is less pronounced (moderate correlation), which can be explained by individual variability in pain sensitivity and the fact that by the 7th to 12th day, while minimal signs of inflammation still persisted, there was no pain.

### 3.2. Histological Analysis

Rabbit oral tissues included teeth and gingiva, underlain by alveolar bone. The mucosa was covered with stratified keratinized epithelium characterized by frequent, short papillae. Beneath the epithelium lay the lamina propria, composed of multidirectional collagen bundles, while the submucosa contained dense connective tissue with blood vessels, adipocytes, muscle fibers and salivary gland ducts.

**Figure 4:**
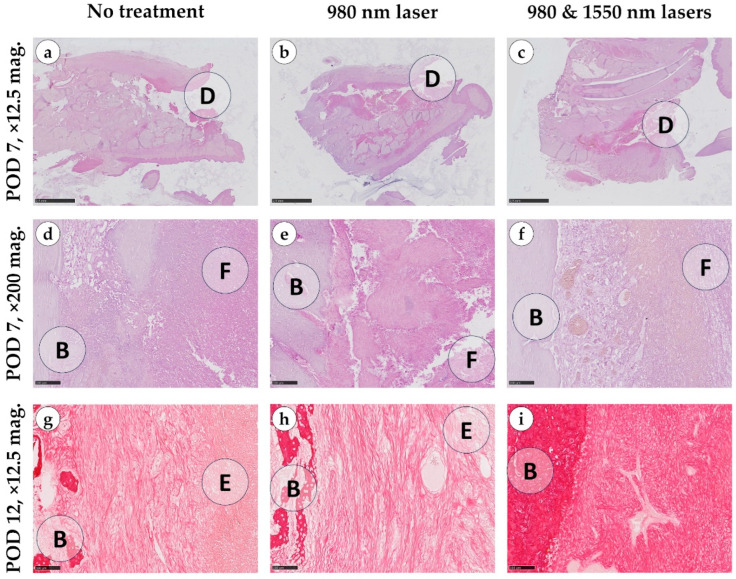
Morphological structure of the alveolar bone of a rabbit at the site of the defect, control group with no treatment (**a**,**d**,**g**), 980 nm laser therapy group (**b**,**e**,**h**) and 980 & 1550 nm laser therapy group (**c**,**f**,**i**), postoperative days (POD) 7 and 12, stained with hematoxylin and eosin (**a**–**f**) and picrosirius red (**g**–**i**), magnifications ×12.5 (scale bar—2.5 mm) and ×200 (scale bar—100 µm). D—area of defect, B—alveolar bone, F—fibrin clot, E—epithelium.

On day 7, the control group exhibited alveolar bone defects measuring 7–10 mm × 2–3 mm, which developed into open, non-epithelialized gingival wounds. The defects, along with adjacent tissues (between alveolar trabeculae and the submucosal layer), were filled with fibrin, erythrocytes, leukocytes, and fragments of necrotic tissue, including small bone particles. Histological findings in the 980 nm laser group were comparable to controls: laser exposure neither altered the vascular response nor increased exudation, hemorrhage or fibrin deposition, and no additional damage attributable to burns or thermal injury was detected (Figure 4).

In contrast, the combination of 980 and 1550 nm lasers induced marked differences. Loose granulation tissue formed between the alveolar bone and the defect center, with abundant thin-walled vessels oriented perpendicularly toward the fibrinous zone. In some samples, early epithelialization of the alveolar bone surface was evident.

By day 12, the overall healing pattern observed earlier in the study persisted. In both the control and 980 nm groups, granulation tissue displayed irregular architecture with heterogeneous compaction arising from the lamina propria and deeper regions of the defect. The regenerating epithelium extended into the socket and partially covered the underlying granulation tissue, which consisted predominantly of thin, loosely arranged collagen bundles with limited directional alignment, giving mostly green and yellow light in polarized light. Despite these morphological changes, the progressive tissue ingrowth did not result in measurable reductions in defect volume.

**Figure A1:**
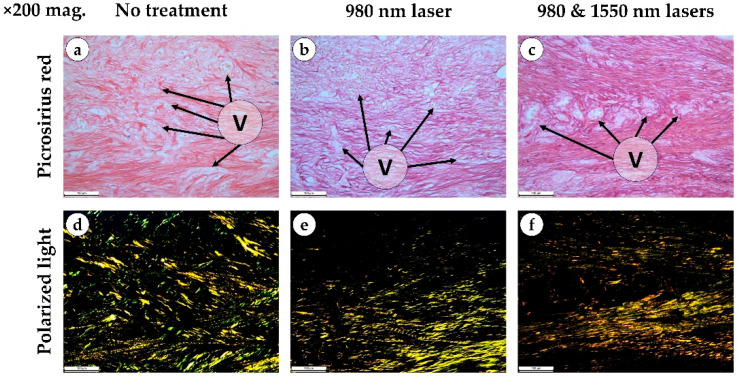
Collagen fiber organization in the granulation tissue surrounding the vascular lumen (V) on day 12 after treatment. (**a**–**c**) Picrosirius red staining at ×200 magnification demonstrates the orientation and density of collagen fibers (black arrows) in the no treatment group (**a**), after 980 nm laser therapy (**b**) and after combined 980 nm + 1550 nm laser therapy (**c**). (**d**–**f**) Corresponding polarized light images highlight birefringent collagen fibers, with brighter yellow–orange signals indicating more mature and aligned collagen bundles. Qualitatively, the dual-wavelength group (**f**) shows more organized and densely packed fibers compared to the other groups, consistent with enhanced maturation of the extracellular matrix. Scale bars—100 µm.

In contrast, the combined 980 nm + 1550 nm laser group demonstrated more advanced extracellular matrix remodeling. Picrosirius red staining revealed denser and more uniformly oriented collagen fibers surrounding the vascular structures, while polarized light microscopy showed brighter, more birefringent (golden and yellow) fiber bundles consistent with greater collagen maturation and organization. These structural improvements were accompanied by a visible reduction in defect volume, and in several specimens, partial fusion of the defect walls indicated early compensation of the socket cavity. Across all groups, bacterial colonies were absent by day 12 (Figure A1).

**Figure 5:**
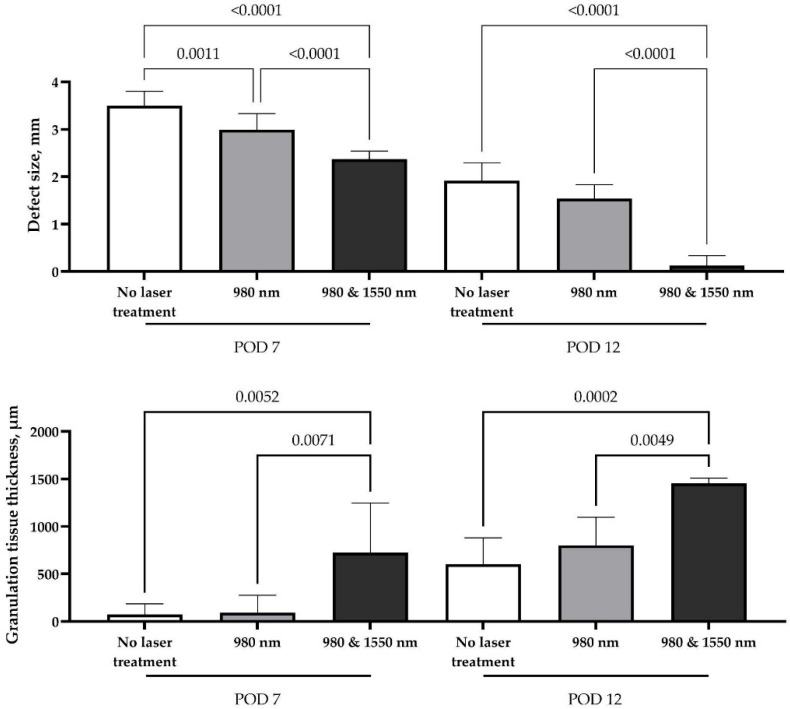
Statistical analysis of macroscopically evaluated defect size and granulation tissue thickness on the wall of the alveolar bone defect, postoperative days (POD) 7 and 12, one-way ANOVA followed by Tukey’s multiple comparison test, mean values ± SD, *p* values less than 0.05 are presented on the graph.

Macroscopic morphometric analysis of defect sizes demonstrated significant differences among all study groups, indicating that laser exposure influenced soft tissue regeneration as early as one week after surgery. By day 7, control wounds showed only partial growth of connective tissue with hyperemic edges and areas of exposed bone. In contrast, the single-wavelength 980 nm group demonstrated more uniform tissue growth with markedly reduced hyperemia, although the defect remained incompletely filled and bone was still partially visible. The most pronounced early effect was observed in the combined double-laser group, where the socket was almost completely filled with newly formed tissue, the margins appeared calm, and no exposed bone could be seen. However, the effect of single-wavelength therapy (980 nm) was transient; by the second week, no significant difference was observed compared with the control group (*p* = 0.2281). At this time point, control sites showed pale granulations, incomplete epithelialization and a persistent depression, while the single-laser wounds resembled conventional healing with partial closure but without full surface recovery. By contrast, complete compensation of the defect volume and full closure with pink mucosa and nearly invisible scar formation were achieved only in the combined double-laser group (Figure 5).

The sustained regenerative effect observed in the double-laser group can be attributed to an accelerated productive phase of wound healing, as confirmed by morphometric analysis. Granulation tissue forming along the defect margins provided a favorable substrate for repair. Its thickness was significantly greater than in both the control and single-laser groups at both examined time points (*p* < 0.01), supporting the enhanced healing response induced by dual-wavelength stimulation.

### 3.3. Microbiological Analysis

**Figure 6:**
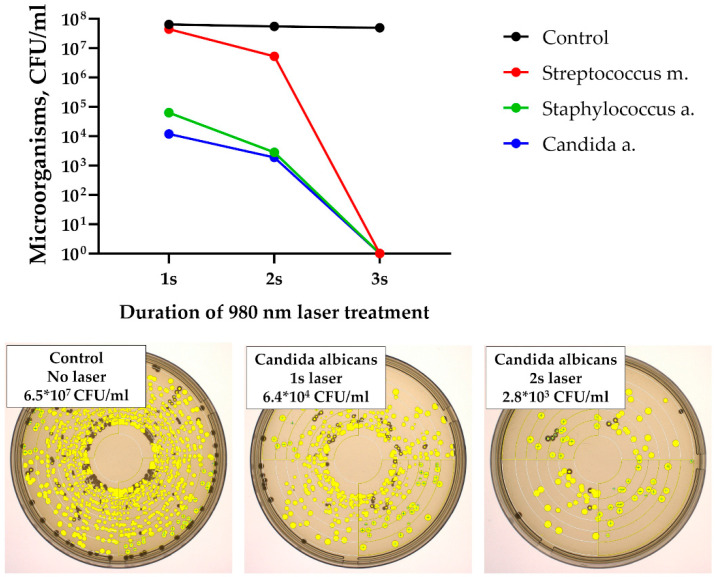
Antibacterial effect of 980 nm laser on cultures of Streptococcus mutans, Staphylococcus aureus, or Candida albicans after 1, 2 and 3 s of exposure. Photographs of microorganisms on Petri dishes were automatically analyzed with the Interscience Scan 4000 automatic colony counter.

In the first stage of the study, exposure of microbial suspensions to laser radiation resulted in a rapid reduction in colony-forming units (CFU). A measurable decline was observed after only 2 s of the treatment, and no microorganisms were identified after 3 s of exposure (Figure 6). No viable Streptococcus mutans, Staphylococcus aureus, or Candida albicans cells were detected beyond this time point.

In the animal study, all groups demonstrated an increase in opportunistic microflora by day 3, coinciding with clinical manifestations of alveolitis (Table 1). In the groups receiving laser therapy, microbial counts decreased sharply thereafter. By day 5, bacterial load was significantly reduced (*p* < 0.001), and by day 7, levels had returned to baseline.

In contrast, animals treated with conventional curettage and antiseptic irrigation maintained a persistently high bacterial load through day 7 (mean 3 × 10^6^ CFU/mL), with no statistically significant change from day 3 values (*p* > 0.05). This correlated with sustained signs of inflammation on both clinical and histological examination. Among the laser-treated animals, the combined laser group (980 nm + 1550 nm) demonstrated the most rapid and pronounced suppression of microbial counts, consistent with improved histological indicators of healing.

## 4. Discussion

The study demonstrates a clear gradation in effectiveness between various alveolitis treatment methods. The most significant positive results were achieved in the combined laser therapy group (980 nm + 1550 nm), suggesting this approach is the most promising.

The obtained data are consistent with the works of other authors, confirming the anti-inflammatory and biostimulating effects of laser therapy in periodontal tissues [9]. The mechanism of action apparently lies in the complex effect: the 980 nm laser, having a pronounced antibacterial effect due to the absorption of radiation by hemoglobin and pigmented microorganisms, effectively sanitizes the socket [10]. At the same time, the 1550 nm laser, penetrating deeper into the tissue and being absorbed by intracellular water, has a modulating effect on cellular metabolism, stimulating the proliferation of fibroblasts and angiogenesis, which accelerates the formation of granulation tissue and epithelialization [11]. The synergistic effect of the combination of these wavelengths accounts for the superior outcomes observed across all evaluated parameters.

The key result is the dynamics of pain syndrome. Rapid pain relief in the combined laser group by day 5 and its complete disappearance by day 7 can be explained not only by reduced hyperemia and swelling, but also by the laser’s direct effect on nerve endings, reducing their sensitivity [12]. In the monotherapy and, especially, mechanical treatment groups, pain syndrome persisted longer, which directly impacts the patient’s quality of life and requires additional medication support.

An important aspect is the difference in swelling dynamics. The fact that swelling in the conventional group did not decrease throughout the entire observation period indicates a persistent, low-grade inflammatory process that was not fully controlled by mechanical curettage and antiseptics. This confirms the limited effectiveness of the standard protocol, and that while it manages the outcome of the tooth extraction, it does not directly address the underlying cause of the inflammation.

The histological examination provided a detailed picture of the reparative processes in the socket of the extracted tooth seven days after the procedure. A critically important result is confirmation of the safety of laser treatment. Laser treatment did not directly create additional detectable defects that could be associated with burns or thermal exposure. No areas of coagulation necrosis, carbonization or other artifacts indicative of thermal damage were detected in any specimen. The most pronounced differences were observed between the combined laser therapy group (980 nm + 1550 nm) and the control group without laser treatment. Combined laser application resulted in the formation of more organized granulation tissue with pronounced angiogenesis (an abundance of thin-walled vessels oriented perpendicular to the fibrin zone). This effect can be explained by the synergistic action of the two wavelengths: while 980 nm radiation provided an antibacterial effect, 1550 nm radiation, absorbed by intracellular water, stimulated fibroblast proliferation and vascular growth [13,14].

The microbiological study demonstrated that laser therapy exerts a strong antimicrobial effect both in vitro and in vivo, with clear advantages over traditional treatment of alveolitis. In vitro, successful eradication of *S. mutans*, *S. aureus*, and *C. albicans* was achieved after only 3 s of exposure, confirming the bactericidal potential of laser treatment through combined thermal and photochemical mechanisms [15]. In vivo, the peak microbial load observed on day 3 corresponded to the acute inflammatory phase, but while surgical curettage and antiseptic irrigation failed to reduce bacterial counts or alleviate inflammation, laser therapy led to a rapid and sustained suppression of pathogenic microflora with recovery to baseline by day 7. Notably, the combination of 980 nm and 1550 nm lasers produced the most pronounced effect, likely reflecting complementary actions: 980 nm contributing to water absorption and hemostasis, and 1550 nm providing deeper penetration, effective biofilm disruption, and stimulation of reparative processes. These findings suggest that the dual action of direct microbial destruction and enhancement of local defense mechanisms makes laser therapy a superior alternative to conventional methods in the management of alveolitis.

Although the microbiological data showed complete eradication of pathogenic microorganisms after only 3 s of laser exposure, the histological analysis revealed that inflammatory cell infiltration and tissue remodeling persisted for several days thereafter. This discrepancy reflects the difference between microbial clearance and the subsequent biological phases of wound healing, which depend on fibroblast activation, angiogenesis and epithelial migration rather than bacterial load alone. Thus, while laser therapy rapidly neutralized the infection, tissue repair followed a delayed but orderly course consistent with normal regeneration dynamics.

This study has limitations related to its use of healthy animal models and short observation period, which may not fully reflect the complexity of human alveolitis or long-term healing outcomes. The microbiological analysis was limited by the absence of log-transformed CFU data and reliance on non-parametric statistics. Additionally, the in vitro bactericidal assay lacked sham-laser controls, which weakened the strength of eradication claims. Although we evaluated whether changes differed between upper and lower jaw sockets, we did not apply formal corrections for multiplicity across endpoints and time points. Nevertheless, the results remained consistent across analyses, and we consider them exploratory. Nevertheless, the findings highlight the translational potential of combining 980 nm and 1550 nm lasers as a minimally invasive, time-efficient approach for socket management. The demonstrated antimicrobial, anti-inflammatory and pro-regenerative effects suggest that dual-laser therapy could be integrated into clinical protocols for alveolitis treatment, pending confirmation in controlled human studies.

## 5. Conclusions

Laser therapy demonstrates notable advantages over conventional approaches in the management of alveolitis by combining antimicrobial effects with stimulation of local defense and reparative processes. In vitro, a clear reduction in microbial growth was observed after short exposure times, while in vivo treatment led to a marked decrease in bacterial load and faster resolution of inflammation compared to traditional curettage and antiseptic irrigation. The combination of 980 nm and 1550 nm wavelengths showed the most consistent improvements across outcomes, suggesting a potential synergistic benefit of their complementary effects. While the sample size was limited, these findings support further exploration of laser-based protocols, particularly dual-wavelength therapy, as a promising strategy for improving clinical outcomes in alveolitis treatment.

## Figures and Tables

**Table 1:** Average number of microorganisms in the oral cavity of rabbits in the experiment at different periods of the study, colony-forming units (CFU).

Group	Before Surgery Mean ± SD	Day 3 Mean ± SD	Day 5 Mean ± SD	Day 7 Mean ± SD
No treatment (n = 6)	1.8 × 10^4^ ± 1.0 × 10^4^	3.3 × 10^6^ ± 2.3 × 10^6^	2.4 × 10^7^ ± 1.6 × 10^7^	3.0 × 10^6^ ± 2.0 × 10^6^
980 nm laser (n = 6)	5.6 × 10^4^ ± 2.5 × 10^4^	5.0 × 10^6^ ± 2.6 × 10^6^	2.0 × 10^5^ ± 4.0 × 10^5^	2.0 × 10^4^ ± 1.0 × 10^4^
Laser 980 nm + 1550 nm (n = 6)	2.0 × 10^4^ ± 0.8 × 10^4^	7.8 × 10^5^ ± 4.0 × 10^5^	4.0 × 10^4^ ± 2.0 × 10^4^	1.0 × 10^4^ ± 1.9 × 10^3^
*p*	>0.05	>0.05	<0.05	<0.05

## Data Availability

The original contributions presented in this study are included in the article. Further inquiries can be directed to the corresponding author.

## Abbreviations

The following abbreviations are used in this manuscript:

CFU	Colony-forming units
ANOVA	Analysis of variance
POD	Post-operative day
